# Potential Role of Carvedilol in the Cardiac Immune Response Induced by Experimental Infection with* Trypanosoma cruzi*

**DOI:** 10.1155/2017/9205062

**Published:** 2017-03-09

**Authors:** Aline Luciano Horta, Ana Luisa Junqueira Leite, G. Paula Costa, Vivian Paulino Figueiredo, André Talvani

**Affiliations:** ^1^Programa de Pós-Graduação em Ciências Biológicas/NUPEB, Universidade Federal de Ouro Preto, Ouro Preto, MG, Brazil; ^2^Departamento de Ciências Biológicas, Universidade Federal de Ouro Preto, Ouro Preto, MG, Brazil; ^3^Programa de Pós-Graduação em Saúde e Nutrição, Universidade Federal de Ouro Preto, Ouro Preto, MG, Brazil; ^4^Programa de Pós-Graduação em Biomas Tropicais, Universidade Federal de Ouro Preto, Ouro Preto, MG, Brazil

## Abstract

*Trypanosoma cruzi* causes a cardiac infection characterized by an inflammatory imbalance that could become the inciting factor of the illness. To this end, we evaluated the role of carvedilol, a beta-blocker with potential immunomodulatory properties, on the immune response in C57BL/6 mice infected with VL-10 strain of* T. cruzi* in the acute phase. Animals (*n* = 40) were grouped: (i) not infected, (ii) infected, (iii) infected + carvedilol, and (iv) not infected + carvedilol. We analyzed parameters related to parasitemia, plasma levels of TNF, IL-10, and CCL2, and cardiac histopathology after the administration of carvedilol for 30 days. We did not observe differences in the maximum peaks of parasitemia in the day of their detection among the groups. The plasma TNF was elevated at 60 days of infection in mice treated or not with carvedilol. However, we observed a decreased CCL2 level and increased IL-10 levels in those infected animals treated with carvedilol, which impacted the reduction of the inflammatory infiltration in cardiac tissue. For this experimental model, carvedilol therapy was not able to alter the levels of circulating parasites but modulates the pattern of CCL2 and IL-10 mediators when the VL10 strain of* T. cruzi* was used in C57BL6 mice.

## 1. Introduction

Chagas disease, although described 108 years ago, remains today as a social and medical problem, affecting nearly 5.7 million people worldwide [1]. Many studies have shown that its pathogenicity goes beyond the* T. cruzi* persistence in muscle tissues, being particularly characterized by an inflammatory imbalance that, despite being crucial for host resistance, also becomes the inciting factor of this illness [2–4]. This immune imbalance can trigger a fierce parasite replication when diminished, but on the other hand, an exacerbated inflammatory response could worsen the host status, by causing irreparable harm to various tissues, leading to organ failure or death [4–6]. Nowadays, benznidazole and nifurtimox are the only antiparasitic drugs lawful for human use [7]. However, both therapies, in addition to their high toxicity, show as well unsatisfactory results when administered in Chagas chronic phase [8–10]. In parallel to the search for compounds with antiparasitic activity, our group has been evaluating the potential immunomodulatory effects of drugs managed in clinical routine to chagasic patients. These therapies (simvastatin, enalapril, losartan, and doxycycline) perform primarily cardiovascular application, but in experimental models and in humans, they also work in the inflammatory response induced by* T. cruzi* [11–13]. Additionally, some of them may also act, directly or indirectly, in the survival of these protozoa [11, 14]. Therefore, in this proposal, we investigated the third-generation beta-blocker, carvedilol, and its potential role in the systemic inflammatory response and in cardiac tissue of mice infected with the VL-10 strain of* T. cruzi*.

## 2. Material and Methods

### 2.1. The VL-10 Strain of the* Trypanosoma cruzi*

The VL-10 strain was first isolated in Minas Gerais, where a female patient by presenting a normal electrocardiogram was classified as asymptomatic [15]. This strain belongs to the TcII DTU of* T. cruzi* [16, 17], previously described as resistant to BZ therapy [18], with cardiac tropism [19, 20], but causing a slight fibrosing inflammation in the C57BL6 mice [11].

### 2.2. Animal Infection and Carvedilol Treatment

We used 40 inbred female C57BL/6 mice, divided into four treatment groups: not infected (NI) (10), infected (I) (10), infected + carvedilol (I + CV) (10), and not infected + carvedilol (NI + CV) (10). The mice were inoculated intraperitoneally with 5 × 10^3^ bloodstream forms of the VL-10 strain of* T. cruzi*, stored in liquid nitrogen, and kept in vivo by consecutive passages in mice of Swiss lineage, in the Laboratory of Immunobiology of Inflammation (LABIIN), at Universidade Federal de Ouro Preto (UFOP). After 24 hours of infection, the carvedilol (15 mg/kg) was diluted in PBS with addition of methyl cellulose for suspension and the therapy was performed for 30 days, by gavage. Untreated animals received the same vehicle in which the drugs were diluted. The C57BL/6 mice were maintained infected for 60 days, and after this period blood collection and euthanasia were carried out to obtain the serum to immune analysis and the cardiac tissue to obtain the cardiac hypertrophy index. For this last parameter, each animal was weighed before being euthanized and the heart was removed and perfused in phosphate saline buffer. After that, the organ was weighed on an analytical balance to establish the ratio of heart weight/body weight (HW/BW).

### 2.3. Parasitological Parameters

The levels of parasitemia were evaluated using 5 *μ*L of blood obtained from the tail vein of infected mice, collected daily to examine living parasites, until the 30th day of infection as previously described by Brener, 1962. The parasitemia curve, the maximum of parasitemia (MP), and the day of maximum parasitemia were analyzed.

### 2.4. Immunoassays

To analyze the interference of carvedilol therapy on inflammatory response associated with* T. cruzi* experimental infection, immunoassays were performed for inflammatory cytokine and chemokine TNF and CCL2/MCP-1 (PeproTech, NJ, USA), respectively, and for the regulatory cytokine IL-10 (PeproTech, NJ, USA). Blood from the orbital venous sinus (0.5 mL) was collected during euthanasia and centrifuged (1500 *g* for 15 minutes at 4°C). The plasma was stored at −80°C. Next, these samples were used to measure TNF, CCL2, and IL-10, according to the protocol recommended by the manufacturer. The samples were simultaneously measured in triplicate.

### 2.5. Morphometric and Histopathological Analysis

Cardiac tissue fragments were fixed in 10% buffered-formalin solution; then, they were dehydrated, cleared, and embedded in paraffin to analyze and quantify the inflammatory infiltration and the amastigote nests. Blocks were cut in 4 mm thick sections and stained in hematoxylin and eosin (HE). Twenty fields from each HE stained section were randomly chosen at 40x magnification, thus totaling 74931 *μ*m^2^, the equivalent area of 50 fields of the analyzed myocardium. Images were obtained in a Leica DM 5000 B micro chamber (Leica Application Suite, UK, version 2.4.0 R1) and processed in the Leica Quinn (V3) image analyzer software. The inflammatory process was assessed through the number of cellular nuclei found in the infected heart tissue and compared to the background of the cardiac cellular nuclei found in the noninfected mice. Amastigote nests were quantified in the Image J 1.45s software, at the National Institute of Health, USA (https://imagej.nih.gov/ij/). The area occupied by parasites was assumed to be the same area previously used to quantify the inflammatory process.

### 2.6. Statistical Analysis

The parameters evaluated in this study were represented by the average of their values and their standard error. We used the Prism program version 5.0 (GraphPad, San Diego, CA, US), where data were analyzed using the Kolmogorov-Smirnov test to confirm patterns of normality. Confirming normality, data were analyzed by one-way ANOVA test for multiple comparisons (Turkey test) and Mann–Whitney test when they were nonparametric.

The relation of CCL2 and IL-10 as predictor of cardiac inflammation induced by the* T. cruzi* infection was performed using the receiver-operator-characteristic (ROC) curve. In brief, the ROC curve is a plot of sensitivity of a test versus its false-positive rate (specificity) for all possible cut points. The area under the curve, ranging from “0” to “1,” provides a measure of the overall accuracy of the test, that is, the ability of the test to correctly classify those with and without the cardiac inflammation. For this analysis, we also used the Prism program version 5.0.

## 3. Results

**Figure 1:**
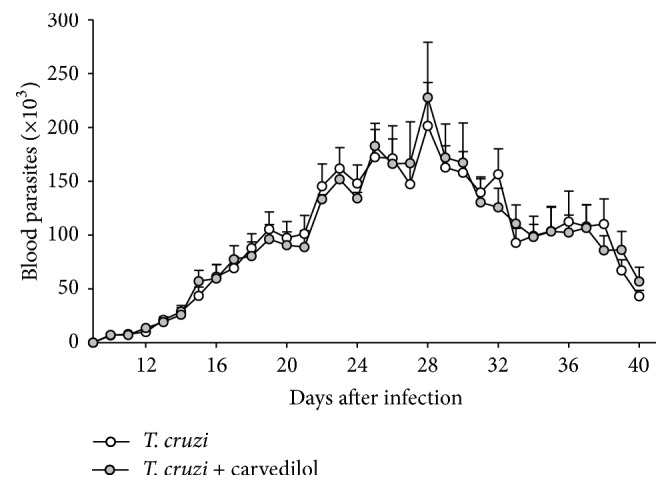
Parasitemia levels after 30 days of oral administration of carvedilol. Parasitemia curve obtained from C57BL/6 mice, infected with 5000 trypomastigote forms of* T. cruzi* VL-10 strain and treated daily with a dose of 15 mg/kg for 30 consecutive days. Each point in the curve represents the daily average of 10 animals.

**Figure 2:**
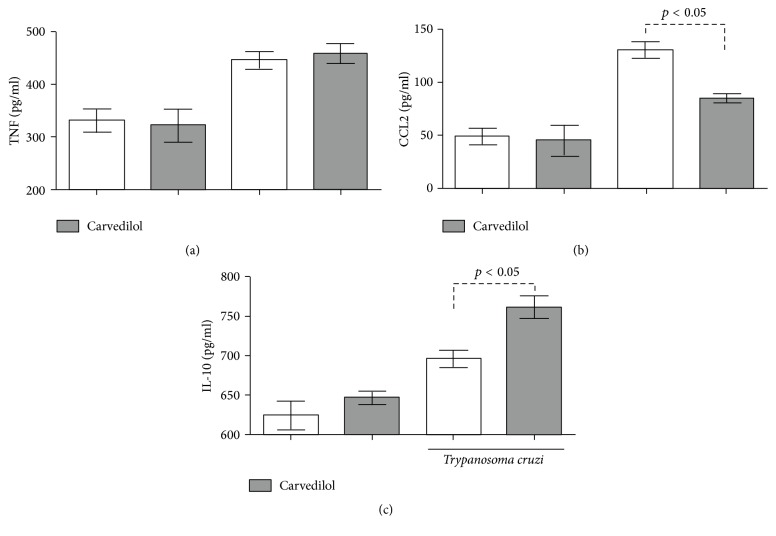
Serum concentration of TNF (a), CCL2 (b), and IL-10 (c) in C57BL/6 mice. The inflammatory and regulatory mediators were evaluated after 30 days of drug intervention and 60 days on infection, in* T. cruzi* infected C57BL/6 mice, treated or not with carvedilol, and noninfected C57BL/6 mice treated or not with the drug. The results are representative of 10 animals/group and expressed as median +/− SEM and *p* < 0.05 indicates difference between infected animals, treated and untreated.

After 30 days of carvedilol therapy and 60 days of infection, we did not observe quantitive differences in the circulating levels of parasites among the groups (Figure 1). There was no differences in the maximum number of blood parasites, in the day where this maximum parasitemia occurred or in the relative heart mass (Table 1). By analyzing the levels of the inflammatory cytokine TNF, an increase was observed in animals infected with the VL-10 strain with significant results (*p* < 0.05) when compared to the noninfected animals under treatment or not (Figure 2(a)). The same pattern was noted with the inflammatory chemokine CCL2 (Figure 2(b)), where we observed an increase in the infected animals when compared to those uninfected. However, the carvedilol reduced the level of circulating CCL2 and increased the regulatory cytokine IL-10 (Figure 2(c)).

**Figure 3:**
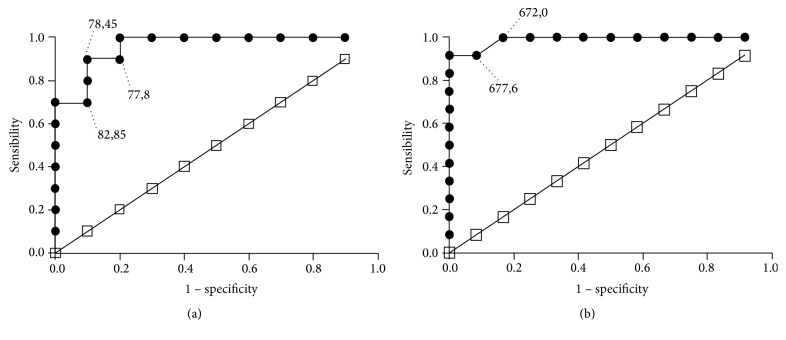
Receiver-operator-characteristic (ROC) curve for the chemokine CCL2 (a) and the regulatory cytokine IL-10 (b) in diagnostic of experimental cardiac inflammation. The ROC curve was assessed for various concentrations of CCL2 and IL-10 in* T. cruzi* infected C57BL/6 mice, under carvedilol treatment or not, during the diagnosis of inflammatory heart disease (CCL2, area under the ROC curve: 0.960 and *p* < 0.0005 and IL-10 area under the ROC curve: 0.989 and *p* < 0.0005).

Both, CCL2 and IL-10, are potential indicators of cardiac tissue inflammation and heart disease in experimental models and human in the last decades.  Figure 3 shows the ROC curve for various concentrations of CCL2 (Figure 3(a)) and IL-10 (Figure 3(b)) in the diagnosis of heart inflammation in the mice studied group pointing the area under the curve with 0.960 ± 0.00051 to CCL2 and 0.989 ± 0.0001, indicating an excellent diagnostic performance. Besides, an elevated concentration of CCL2 presented a sensitivity of 76% and specificity of 80%, while the IL-10 shows a sensitivity of 90% and specificity of 91%.

**Figure 4:**
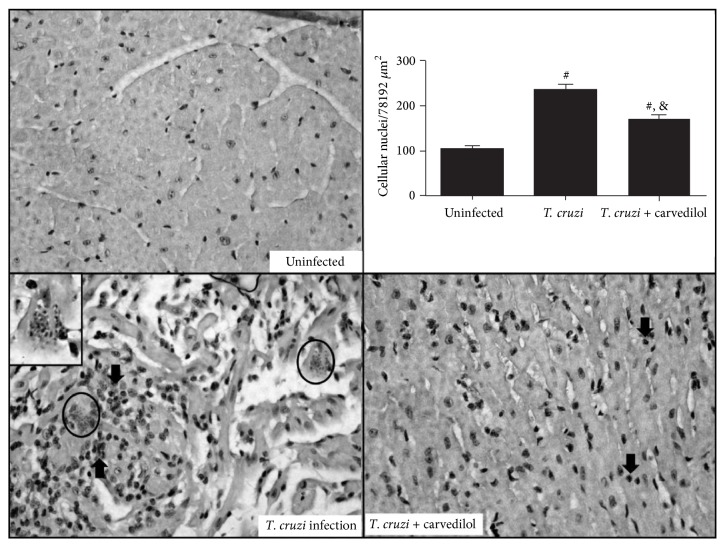
Carvedilol reduces inflammation in the* T. cruzi* infected cardiac tissue. The hearts from C57BL6 mice infected with* T. cruzi* were extracted after 30 days of infection/treatment and the leukocyte infiltration was evaluated by H&E. The noninfected animals were evaluated in parallel and sections were quantified in sections with 40x of magnification. Data are representative of the groups (*n* = 10 animals) and ^#^*p* < 0.05 in relation to the noninfected group; ^&^*p* < 0.05 in relation to the infected group without treatment.

Regarding this present study, the VL-10 strain of the* T. cruzi* was associated with a moderate mononuclear cell influx, which reduced under pharmacological therapy with carvedilol (Figure 4) when quantified in a total area of 74931 *μ*m^2^. In addition, the area occupied by amastigote nests of the parasite in the cardiac tissue was lower and quantified only in the infected group (mean: 0.04 *μ*m ± SE: 0.017), which constitute an intrinsic characteristic of the VL-10 strain of this parasite in mice.

## 4. Discussion

The third generation of beta-blocker, the carvedilol, has acquired a prominent role in different types of treatments such as antihypertensive [21], heart failure [22], and thrombosis [23], in addition to its high antioxidant activity [24], and improvements were made in left ventricular ejection fractions [25–27]. Some studies have shown that carvedilol besides the previously described and widely discussed effects also displays an immunomodulatory activity [26, 28, 29]. de Araújo Júnior and coworkers (2013) also showed this, by suggesting that this treatment is able to reduce the production of proinflammatory cytokines such as IL1*β* and TNF and inhibiting matrix metalloproteinases 2 and 9 in rodents. Other studies also reported that carvedilol can reduce serum TNF production and is able to suppress the CD107a and HLA-DR on circulating cytotoxic T cells, all compounds critical to the pathogenesis of chronic heart failure [23, 30–32]. Besides, this beta-blocker also interferes with intracellular signaling proteins (e.g., NF-*κ*B, Src-ERK) and with the expression of chemokines (e.g., CCL2) in experimental and noncardiac study models [33, 34].

In this present study, we did not observe reduction in the circulating TNF under carvedilol therapy when the evaluation was done at 60 days of infection. However, this critical cytokine is described to activate and coordinate, in part, the cardiac inflammatory response in the murine model of* T. cruzi* infection as well as in human Chagas disease [2, 4]. In this inflammatory context, the presence of inflammatory cytokines such as TNF and IFN-gamma is a trigger to different chemokines expression in the mononuclear and cardiac cells from animals infected with* T. cruzi* [35]. Chemokines are responsible for the recruitment of leukocytes into the infected tissue coordinating the magnificence of the inflammatory process. Genes from chemokines involved in monocyte chemotaxis (e.g., CCL2, CCL3, and CCL5), as well as their respective receptors, were more expressed and/or detected in heart from human and experimental animals infected with* T. cruzi* [13, 36–38].

The monocyte chemoattractant protein-1 (CCL2) acts on resident and recruited macrophages/monocytes inducing NF-kB activation in these cells which can interact with other inflammatory cells potentiating the chronic inflammation in the infected cardiac tissue [13, 38]. The CCL2 has also been pointed out as a potential biomarker of cardiomyopathy in experimental and human* T. cruzi* infection [11–14, 36, 38, 39]. Here, we observed that carvedilol therapy was associated with the reduction of CCL2 in infected animals and this data matched with the reduction of the leukocyte infiltration into the cardiac tissues. Previously, a clinical benefit of this beta-blocker in association with enalapril and spironolactone was described in chronic chagasic patients inducing an increase in the left ventricle ejection fraction and a reduction in the circulating levels of the chemokine CCL5 and the brain natriuretic peptide [26]. Other clinical studies focused on the role of the carvedilol potentiating the antioxidative effect induced by C and E vitamins in chagasic individuals [40, 41]. Even considering the oxidative stress as part of the general inflammatory process, there a very few clinical and experimental studies involving carvedilol and mononuclear cells infiltration or the inflammatory mediators release during the* T. cruzi* infection.

Conversely, the propose of these inflammatory cytokines/chemokines released is the activation and recruitment of the new monocytes or T cells from circulation into the cardiac tissue, aiming at the elimination of the amastigote forms of the parasite. This crosstalk between cells and inflammatory mediators defines the chronic status of the pathogenesis and is, in part, regulated by the regulatory cytokines such as IL-10. The IL-10 has also been described to lead to the regulation of the immune response in those asymptomatic individuals with* T. cruzi* infection [42]. In the present study, we observed that animals treated with carvedilol released high levels of plasma IL-10, possibly suggesting its modulatory effect since these data were followed by a low production of CCL2 and low migration of leukocytes into cardiac tissue. Herewith, both CCL2 and IL-10 were suggested as an excellent diagnostic performance in detection of the cardiac inflammation induced by the* T. cruzi* (area under the ROC curve above 0.9). Therefore, the TNF, CCL2, and IL-10 were chosen here as markers to the carvedilol treatment due to their importance in distinct studies involving* T. cruzi* infection [2, 6]; however all the responses observed behind the* T. cruzi* infection possess more complexity than they seem.

It is noteworthy that the animal lineage chosen for this study as well as the parasite strain exerts interference in the profile of the inflammatory response. Previously, our group demonstrated a high tissue inflammation and a higher plasma production of cytokines and chemokines (TNF, IFN-g, CCL2, and CCL5) in C57BL6 mice infected with an inflammatory strain of* T. cruzi* (e.g., Colombian) [11, 43]. More recently, we investigated C57BL6 mice infected with the VL-10 of* T. cruzi* and observed a low tissue inflammation, an absence of parasites in the cardiac tissue observed by optical microscopic analysis, and a reduction of the plasma TNF, CCL2, and CCL5 chemokines [12]. In other words, in this present study we also used the VL-10 strain of the parasite, a benznidazole-resistant strain that induces lower mortality in animals and, thus, less inflammation. And in this way, any inflammatory alteration caused by pharmacological intervention represents a significant scientific finding. Some not so expressive data negatively observed under carvedilol intervention could have been intensely observed with a change in the lineage (parasites and hosts) model picked.

In summary, we observed that carvedilol treatment was not able to alter the levels of circulating parasites but changed the patterns of circulating levels of the regulatory cytokine IL-10 and the chemokine CCL2. Besides, other studies show that the VL-10 strain of* T. cruzi* can induce high inflammation levels in different animal lineages [44, 45], such as the Swiss mice and dogs; the C57BL/6 mice lineage shows low inflammation levels and low parasitic burden. Even so, carvedilol therapy during acute phase of infection was able to modulate the CCL2 and intensify the production of the regulatory cytokine IL-10 in serum from infected mice. Hence, based on our previous experience with the VL-10 [12], the profile of these inflammatory mediators during acute phase of infection can reflect part of the pathogenesis in the chronic stage, including reducing cardiac damage enzymes such as CK and CK-MB. New studies concerning this therapy in different animal lineages and in human are essential to understand the pleiotropic effects of this drug on the immune response and its potential protective role in the pathogenesis of heart disease triggered by* T. cruzi*.

## Figures and Tables

**Table 1:** Mean of biological parameters obtained in C57BL/6 mice *T. cruzi* infected or not, submitted to carvedilol intervention (15 mg/kg/day for 30 consecutive days).

Parameters	Groups			
	Infected + carvedilol	Infected	Not infected + carvedilol	Not infected
Maximum of the parasitemia (MP) (mean of 10 mice)	227,8 ± 154,3	201,4 ± 127,6	—	—
Day of the MP	26,9 ± 2,470	28,2 ± 3,795	—	—
Relative heart weight (mg/g)	0,0047 ± 0,0015	0,0042 ± 0,0003	0,0046 ± 0,0004	0,0055 ± 0,0012

No statistical difference was observed in any of the parameters evaluated above.

## References

[1] Tanowitz H. B., Machado F. S., Spray D. C. (2015). Developments in the management of Chagas cardiomyopathy. *Expert Review of Cardiovascular Therapy*.

[2] Teixeira M. M., Gazzinelli R. T., Silva J. S. (2002). Chemokines, inflammation and Trypanosoma cruzi infection. *Trends in Parasitology*.

[3] Sherbuk J. E., Okamoto E. E., Marks M. A. (2015). Biomarkers and mortality in severe chagas cardiomyopathy. *Global Heart*.

[4] Guedes P. M. M., Andrade C. M. D., Nunes D. F. (2016). Inflammation enhances the risks of stroke and death in chronic chagas disease patients. *PLoS Neglected Tropical Diseases*.

[5] Andrade Z. A. (1985). A patologia da doença de Chagas no homem. *Annales de la Societe Belge de Medecine Tropicale*.

[6] Talvani A., Teixeira M. M. (2011). Inflammation and chagas disease: some mechanisms and relevance. *Advances in Parasitology*.

[7] Pérez-Molina J. A., Perez A. M., Norman F. F., Monge-Maillo B., López-Vélez R. (2015). Old and new challenges in chagas disease. *The Lancet Infectious Diseases*.

[8] Oliveira M. d., Nagao-Dias A. T., Oliveira de Pontes V. M., Souza Júnior A. S., Luna Coelho H. L., Branco Coelho I. C. (2008). Tratamento etiológico da doença de Chagas no Brasil. *Revista de Patologia Tropical*.

[9] Santos F. M., Lima W. G., Gravel A. S. (2012). Cardiomyopathy prognosis after benznidazole treatment in chronic canine Chagas' disease. *Journal of Antimicrobial Chemotherapy*.

[10] Morillo C. A., Marin-Neto J. A., Avezum A. (2015). Randomized trial of benznidazole for chronic chagas' cardiomyopathy. *New England Journal of Medicine*.

[11] Silva R. R., Shrestha-Bajracharya D., Almeida-Leite C. M., Leite R., Bahia M. T., Talvani A. (2012). Short-term therapy with simvastatin reduces inflammatory mediators and heart inflammation during the acute phase of experimental Chagas disease. *Memórias do Instituto Oswaldo Cruz*.

[12] Penitente A. R., Leite A. L. J., Costa G. D. P. (2015). Enalapril in combination with benznidazole reduces cardiac inflammation and creatine kinases in mice chronically infected with trypanosoma cruzi. *American Journal of Tropical Medicine and Hygiene*.

[13] Paula Costa G. d., Lopes L. R., Silva M. C. (2016). Doxycycline and benznidazole reduce the profile of Th1, Th2, and Th17 chemokines and chemokine receptors in cardiac tissue from chronic *Trypanosoma cruzi*-infected dogs. *Mediators of Inflammation*.

[14] Melo L., Caldas I. S., Azevedo M. A. (2011). Low doses of simvastatin therapy ameliorate cardiac inflammatory remodeling in Trypanosoma cruzi-infected dogs. *American Journal of Tropical Medicine and Hygiene*.

[15] Martins C., Reis-Cunha J. L., Silva M. N. (2011). Identification of genes encoding hypothetical proteins in open-reading frame expressed sequence tags from mammalian stages of Trypanosoma cruzi. *Genetics and Molecular Research*.

[16] Baptista C. S., Vêncio R. Z. N., Abdala S. (2006). Differential transcription profiles in *Trypanosoma cruzi* associated with clinical forms of Chagas disease: maxicircle NADH dehydrogenase subunit 7 gene truncation in asymptomatic patient isolates. *Molecular and Biochemical Parasitology*.

[17] Moreno M., D'ávila D. A., Silva M. N. (2010). Trypanosoma cruzi benznidazole susceptibility in vitro does not predict the therapeutic outcome of human Chagas disease. *Memorias do Instituto Oswaldo Cruz*.

[18] Caldas I. S., Talvani A., Caldas S. (2008). Benznidazole therapy during acute phase of Chagas disease reduces parasite load but does not prevent chronic cardiac lesions. *Parasitology Research*.

[19] Vago A. R., Macedo A. M., Oliveira R. P. (1996). Kinetoplast DNA signatures of Trypanosoma cruzi strains obtained directly from infected tissues. *American Journal of Pathology*.

[20] Dvorak J. A. (1984). The natural heterogeneity of trypanosoma cruzi: biological and medical implications. *Journal of Cellular Biochemistry*.

[21] Leonetti G., Egan C. G. (2012). Use of carvedilol in hypertension: an update. *Vascular Health and Risk Management*.

[22] Prabhu S. D., Chandrasekar B., Murray D. R., Freeman G. L. (2000). *β*-Adrenergic blockade in developing heart failure: effects on myocardial inflammatory cytokines, nitric oxide, and remodeling. *Circulation*.

[23] Lin P.-Y., Shen H.-C., Chen C.-J. (2010). The inhibition in tumor necrosis factor-*α*-induced attenuation in endothelial thrombomodulin expression by carvedilol is mediated by nuclear factor-*κ*B and reactive oxygen species. *Journal of Thrombosis and Thrombolysis*.

[24] Miranda C. P., Botoni F. A., Rocha M. O. D. C. (2014). Immunopharmacological approach of carvedilol in chronic chagas heart disease. *Arquivos Brasileiros de Cardiologia*.

[25] Gerson M. C., Craft L. L., McGuire N., Suresh D. P., Abraham W. T., Wagoner L. E. (2002). Carvedilol improves left ventricular function in heart falure patients with idiopathic dilated cardiomyopathy and a wide range of sympathetic nervous system function as measured by iodine 123 metaiodobenzylguanidine. *Journal of Nuclear Cardiology*.

[26] Botoni F. A., Poole-Wilson P. A., Ribeiro A. L. P. (2007). A randomized trial of carvedilol after renin-angiotensin system inhibition in chronic Chagas cardiomyopathy. *American Heart Journal*.

[27] Pauschinger M., Rutschow S., Chandrasekharan K. (2005). Carvedilol improves left ventricular function in murine coxsackievirus-induced acute myocarditis Association with reduced myocardial interleukin-1*β* and MMP-8 expression and a modulated immune response. *European Journal of Heart Failure*.

[28] El Desoky E. S. (2011). Drug therapy of heart failure: an immunologic view. *American Journal of Therapeutics*.

[29] de Araújo Júnior R. F., Souza T. O., de Medeiros C. A. X. (2013). Carvedilol decrease IL-1*β* and TNF-*α*, inhibits MMP-2, MMP-9, COX-2, and RANKL expression, and up-regulates OPG in a rat model of periodontitis. *PLoS ONE*.

[30] Ohtsuka T., Hamada M., Hiasa G. (2001). Effect of beta-blockers on circulating levels of inflammatory and anti-inflammatory cytokines in patients with dilated cardiomyopathy. *Journal of the American College of Cardiology*.

[31] Li B., Liao Y.-H., Cheng X., Ge H., Guo H., Wang M. (2006). Effects of carvedilol on cardiac cytokines expression and remodeling in rat with acute myocardial infarction. *International Journal of Cardiology*.

[32] Shaw S. M., Coppinger T., Waywell C. (2009). The effect of beta-blockers on the adaptive immune system in chronic heart failure. *Cardiovascular Therapeutics*.

[33] Ding Q., Tian X.-G., Li Y., Wang Q.-Z., Zhang C.-Q. (2015). Carvedilol may attenuate liver cirrhosis by inhibiting angiogenesis through the VEGF-Src-ERK signaling pathway. *World Journal of Gastroenterology*.

[34] Amirshahrokhi K., Khalili A. (2016). Carvedilol attenuates paraquat-induced lung injury by inhibition of proinflammatory cytokines, chemokine MCP-1, NF-*κ*B activation and oxidative stress mediators. *Cytokine*.

[35] Vasconcelos R. H. T., Azevedo E. A. N., Diniz G. T. N. (2015). Interleukin-10 and tumour necrosis factor-alpha serum levels in chronic Chagas disease patients. *Parasite Immunology*.

[36] Talvani A., Rocha M. O. C., Barcelos L. S., Gomes Y. M., Ribeiro A. L., Teixeira M. M. (2004). Elevated concentrations of CCL2 and tumor necrosis factor—*α* in chagasic cardiomyopathy. *Clinical Infectious Diseases*.

[37] Roffê E., Oliveira F., Souza A. L. S. (2010). Role of CCL3/MIP-1*α* and CCL5/RANTES during acute *Trypanosoma cruzi* infection in rats. *Microbes and Infection*.

[38] Paiva C. N., Figueiredo R. T., Kroll-Palhares K. (2009). CCL2/MCP-1 controls parasite burden, cell infiltration, and mononuclear activation during acute Trypanosoma cruzi infection. *Journal of Leukocyte Biology*.

[39] Ramasawmy R., Cunha-Neto E., Faé K. C. (2006). The monocyte chemoattractant protein-1 gene polymorphism is associated with cardiomyopathy in human Chagas disease. *Clinical Infectious Diseases*.

[40] Budni P., Pedrosa R. C., Garlet T. R. (2012). Carvedilol attenuates oxidative stress in chronic chagasic cardiomyopathy. *Arquivos Brasileiros de Cardiologia*.

[41] Budni P., Pedrosa R. C., Dalmarco E. M., Dalmarco J. B., Frode T. S., Filho D. W. (2013). Carvedilol enhances the antioxidant effect of vitamins E and C in chronic Chagas heart disease. *Arquivos Brasileiros de Cardiologia*.

[42] Gomes J. A. S., Bahia-Oliveira L. M. G., Rocha M. O. C., Martins-Filho O. A., Gazzinelli G., Correa-Oliveira R. (2003). Evidence that development of severe cardiomyopathy in human Chagas' disease is due to a Th1-specific immune response. *Infection and Immunity*.

[43] Magalhães L. M. D., Viana A., Chiari E., Galvão L. M. C., Gollob K. J., Dutra W. O. (2015). Differential activation of human monocytes and lymphocytes by distinct strains of Trypanosoma cruzi. *PLoS Neglected Tropical Diseases*.

[44] Caldas I. S., da Matta Guedes P. M., dos Santos F. M. (2013). Myocardial scars correlate with eletrocardiographic changes in chronic Trypanosoma cruzi infection for dogs treated with Benznidazole. *Tropical Medicine and International Health*.

[45] Higyno P. M. S., Mendes P. F., de Miranda M. B. (2015). Vasoactive intestinal peptide reduces the inflammatory profile in mice infected with *Trypanosoma cruzi*. *Experimental Parasitology*.

